# Virtual screening and molecular dynamics simulations identify repurposed drugs as potent inhibitors of Histone deacetylase 1: Implication in cancer therapeutics

**DOI:** 10.1371/journal.pone.0316343

**Published:** 2025-01-03

**Authors:** Mohammed Alrouji, Sabina Yasmin, Mohammed S. Alshammari, Fahad A. Alhumaydhi, Sharaf E. Sharaf, Moyad Shahwan, Anas Shamsi

**Affiliations:** 1 Department of Medical Laboratories, College of Applied Medical Sciences, Shaqra University, Shaqra, Saudi Arabia; 2 Department of Pharmaceutical Chemistry, College of Pharmacy, King Khalid University, Abha, Saudi Arabia; 3 Department of Clinical Laboratory Sciences, College of Applied Medical Sciences, Shaqra University, Shaqra, Saudi Arabia; 4 Department of Medical Laboratories, College of Applied Medical Sciences, Qassim University, Buraydah, Saudi Arabia; 5 Pharmaceutical Sciences Department, College of Pharmacy, Umm Al-Qura University, Makkah, Saudi Arabia; 6 Department of Clinical Sciences, College of Pharmacy and Health Sciences, Ajman University, Ajman, United Arab Emirates; 7 Center of Medical and Bio-Allied Health Sciences Research (CMBHSR), Ajman University, Ajman, United Arab Emirates; Instituto Politecnico Nacional, MEXICO

## Abstract

Epigenetic processes are the critical events in carcinogenesis. Histone modification plays a crucial role in gene expression regulation, where histone deacetylases (HDACs) are key players in epigenetic processes. Inhibiting HDACs has shown promise in modern cancer therapy. However, the non-selective nature and drug resistance of most HDAC inhibitors (HDACIs) limits their clinical use. This limitation prompts a search for isoform-selective and more effective inhibitors. Histone deacetylase 1 (HDAC1) is a member of the class I HDAC family and has emerged as a promising target in various diseases, including cancer and neurodegeneration. Drug repurposing has gained significant interest in identifying treatments for new targets, which involves finding new uses for existing drugs beyond their original medical indications. Here, we employed virtual screening of repurposed drugs from the DrugBank database to identify potential HDAC1 inhibitors. We conducted a series of analyses, including molecular docking, drug profiling, PASS evaluation, and interaction analysis. Molecular dynamics (MD) simulations and MM-PBSA analysis were also performed for 300 ns. Through these analyses, we pinpointed Alectinib, which exhibits a promising drug profile in PASS analysis and higher affinity and efficiency for HDAC1 than the reference inhibitor. MD simulations revealed that Alectinib stabilizes HDAC1 with minimal structural perturbations. The findings suggest that Alectinib holds promise as a therapeutic lead for HDAC1-associated carcinogenesis after required validation.

## 1. Introduction

Cancer is a complex and multifactorial disease that remains one of the leading causes of mortality worldwide [1]. The cancer incidence rises steadily across modern populations. Even though significant progress has been made in studying the molecular mechanisms of cancer, the management of this disease remains a substantial challenge [2]. One of the distinctive features of cancer is the violation of epigenetic processes [3]. These processes influence the patterns of gene expression without changing the DNA sequence of the gene in question [4]. Of these epigenetic mechanisms, histone acetylation is deemed to be one of the most crucial functions [5]. This process is controlled by the balance of histone acetyltransferases (HATs) and histone deacetylases (HDACs) [6]. It has a function in chromatin structure and gene regulation [7]. HDACs are a group of enzymes that remove the acetyl group from histone proteins that have been recognized to play the role of regulating genes [8]. Furthermore, HDACs are involved in various physiological and pathophysiological processes, such as carcinogenesis [9].

Aberrant expression and activity of HDACs have been associated with the onset and progression of different cancers, making them ideal for therapeutic modulation [10]. HDAC inhibitors (HDACIs) have been of interest in the development of anticancer drugs [11]. They can influence gene expression profiles in carcinogenesis by promoting cell cycle arrest, apoptosis, and suppressing angiogenesis [12]. However, the clinical application of the current HDACIs is limited by their off-target effects and resistance to drugs [13]. This results in undesirable side effects and a limiting toxic dose [14]. To address these challenges, there is increasing focus on the development of isoform-selective HDACIs. These are intended to selectively block some of the HDAC isoforms that are implicated in the disease process. Of these isoforms, Histone deacetylase 1 (HDAC1) has been determined to hold great potential of being a target [15]. It has important roles in many cell processes, such as cell division, cell growth, and cell death and is associated with the development of cancer and its metastasis [16].

Drug repurposing is the process of identifying new therapeutic applications of drugs that have been approved for different targets and diseases [17]. This approach has attracted considerable attention for discovering treatments for new targets because of several advantages that include shorter time and cost of development, well-tolerated safety profiles, multiple modes of action, and meeting unmet medical needs [18]. Approaches employed in drug repurposing include several computational techniques such as virtual screening and molecular dynamics (MD) simulations [19]. These approaches utilize a range of methods for the discovery and fine-tuning of novel inhibitors for a predefined target. It enables high-throughput screening of large chemical databases to find compounds with the desired pharmacological activity. MD simulations are useful in understanding the dynamic characteristics of protein-ligand complexes [20]. They also give a clue about their stability within a given period.

In the present study, we aim to identify potential HDAC1 inhibitors among repurposed drugs through an integrated virtual screening protocol involving molecular docking and all-atom MD simulations followed by essential dynamics analyses. Using the DrugBank database, we performed an integrated structure-guided screening to find compounds with high binding affinity to the HDAC1 binding pocket. The next step was drug profiling, PASS evaluation, and interaction analysis to select promising hits for further investigation. All-atom MD simulations were performed to estimate the stability and dynamic properties of the compounds interacting with HDAC1 and to understand the binding modes. The outcomes from this study might be useful for the identification and development of repurposed HDAC1 inhibitors with promising affinity towards the target protein and acceptable efficacy in the treatment of diseases associated with HDAC1, including cancer and neurodegenerative diseases. This makes this approach cheap and faster than the normal drug discovery process and may bring these findings to clinical settings faster.

## 2. Materials and methods

### 2.1. Computational resources

The computations were performed using an HP Z840 server, and the power supply to the server was kept constant for the study. Molecular docking screening was performed by using several programs like MGL AutoDock [21] and InstaDock [22]. All structural analysis and visualization of the molecular complexes were done using PyMOL [23] and Discovery Studio Visualizer [24]. Concerning the identified sources of data, different web-based resources and databases were used for data collection and analysis. The RCSB Protein Data Bank (PDB) was exploited for structural details of the target HDAC1 [25, 26]. The DrugBank database helped in the use of the repurposed drugs library for screening [27]. Moreover, for drug profiling and biological activity predictions, the Prediction of Activity Spectra for Substances (PASS) server was used [28]. Altogether, these resources provided computational support for the workflow that allowed for the analyses. The MD simulations were carried out in the GROMACS simulation suite [29] for all-atom modeling.

### 2.2. Receptor and library preparation

The X-ray crystal structure of human HDAC1 was retrieved from the RCSB Protein Data Bank (PDB) in PDB format (ID: 5ICN, determined at 3.30Å) [26]. Water molecules and heteroatoms, including co-crystallized ligands, were removed to prepare protein structure for virtual screening. Some of the residues were absent in the crystal structure of the protein. Homology modeling was performed using MODELLER (version 10) software using PyMod-3 embedded in PyMOL [30]. The missing residues were filled by employing parent coordinates from the self-template. This ensured the completeness and accuracy of the HDAC1 structure for subsequent analyses. The structure was further optimized with the help of the MGL AutoDock and InstaDock programs. The DrugBank database was utilized to repurpose the drug library. The library was refined for any biologics and unnecessary molecules. The structures were converted to the PDBQT format, optimized for docking by assigning Gasteiger charges, and energy minimized using InstaDock v1.2 to prepare them for docking and subsequent simulations. The refined library was utilized for the docking screening with HDAC1.

### 2.3. Molecular docking screening

Molecular docking screening is an important step in the drug discovery and development process, offering an effective way to screen vast databases of small-molecule compounds against protein targets [31]. It encompasses the prediction of how receptors will engage with small molecules. It is to estimate the position and orientation of ligands when they interact with a protein. Since the number of compounds to be analyzed is large, the time factor is very crucial in the docking process. For this purpose, molecular docking-based virtual screening was performed to screen HDAC1 inhibitors from the pool of drugs that are available for docking calculations. InstaDock was used as a docking tool because of the speed with which it worked. The docking screening was performed blindly, and all the ligands were free to map the whole structure of the protein. This aided molecules to identify the right binding sites on HDAC1 that would be ideal to bind with. The docking process was then finished, and the output was examined using the log and out files. These top hits were obtained using the criteria of their high-affinity scores with HDAC1. This suggested the extent of binding between the compounds and the target protein.

### 2.4. Drug profiling and PASS evaluation

Drug profiling is a crucial step in drug development which is used to characterize the drug’s properties, behavior, and effects [32]. The prediction of activity spectra for substances (PASS) analysis serves as another valuable strategy for evaluating chemical-biological interactions [28]. It also assesses the biological properties of chemical compounds. In this study, we employed the PASS server to analyze the biological properties of screened compounds. The PASS server uses an internal algorithm that employs molecular fragments of chemical compounds as descriptors. This approach recommends biological properties for input compounds based on structural features and similarity to known compounds with annotated activities. The analysis yielded two descriptors, i.e., the ’probability to be active (Pa)’ and the ’probability of being inactive (Pi)’. A higher Pa value indicates a higher likelihood of the corresponding property for the compound. Conversely, a higher Pi value suggests a higher probability of inactivity.

### 2.5. Interaction analysis

To analyze the interaction and to know the binding mechanisms of the docked protein-ligand complexes, an interaction analysis was performed. This analysis was performed to define all binding poses and to find all possible contacts between the protein and ligand molecules. PyMOL and Discovery Studio Visualizer were used for visual analysis and interaction studies of the protein-ligand complexes. Within PyMOL, interactions within the distance of 3.5 Å within the protein-ligand complex were labeled. This aided in the identification of key interactions in proximity. Meanwhile, Discovery Studio Visualizer provided detailed information on the type of interactions, as well as the participating residues and atomic coordinates. More specifically, the contacts at the active site and the binding site of HDAC1 were of interest in this case. Only compounds with specific interactions with these critical residues were chosen for the subsequent analyses. Furthermore, to confirm the results of the docking and to assess the accuracy of the predicted binding interactions, the binding of one of the known HDAC1 binding partners was used as a reference.

### 2.6. MD simulations protocol

The MD simulations were carried out using the GROMACS 2020 beta package. These were meant to study the temporal characteristics of free HDAC1 and its complexes in the selected ligands. The initial conformations of HDAC1 and docked ligands were obtained from the docking study. For the receptor protein, the GROMOS 54A7 force field was employed. The topological analysis of the ligands and the atomic charges was done using the ATB server. The leap module was used to build the topology files. Coordinate files were also generated using PyMOL for the complex systems. The solvation was performed with the help of TIP3P water model within the virtual box. An appropriate number of counterions were added to neutralize the systems. The SHAKE algorithm was used for hydrogen bonds. The electrostatic interactions of long-range were handled by the particle mesh Ewald (PME) method. All systems’ energy minimization was done with 10000 steps of the steepest descent algorithm. Both systems were warmed up from 0 to 300 K at the rate of 100 ps. This was then proceeded by equilibration for 1000 dynamics steps at 300 K under constant pressure. A production run of 300 ns for each system was performed. The run kept the temperature and pressure of the reaction constant. All the trajectories obtained from the MD simulations were processed using the gmx modules available in GROMACS. The included parameters were root-mean-square deviation (RMSD), root-mean-square fluctuation (RMSF), radius of gyration (*R*g), solvent-accessible surface area (SASA), and hydrogen bonds.

### 2.7. Principal component analysis

Principal component analysis (PCA) is a very effective method widely applied for studying the patterns of motion in proteins [33]. In PCA, the coordinates of *C*α atoms were used. These coordinates produce a covariance matrix (*C*) that defines how the atomic coordinates are related and correlated. The PCA was also carried out from the simulated trajectories of the HDAC1 and its complexes with the elucidated compounds. While applying the diagonalization on the covariance matrix, eigenvalues and eigenvectors were determined. Eigenvectors are the principal components (PCs) of motion. Each eigenvector can be interpreted as a certain mode of motion or conformational transition in the protein. Generally, the first few eigenvectors contain most of the variance information of the data. These are referred to as the PCs and are of main concern. The positional covariance matrix *C* can be generated as:

$$C_{i}=<(q_{i}-<q_{i}>)(q_{j}-<q_{j}>)>\;(i,j=1,2,\ldots ,3N)$$

where q_i_ and q_j_ signify the cartesian coordinates for the i^th^, j^th^ position of the C_α_ atom and N is the number of C_α_ atoms.

### 2.8. Free energy landscapes

Free energy landscape (FEL) analysis is another useful tool in computational biophysics and structural biology, providing a detailed understanding of protein stability, folding, and function [33]. FEL analysis maps the energy landscape of a protein system by using collective variables that define the conformational space of the protein, and thus it establishes low-energy conformations and the pathways that connect them. The FELs were constructed as follows:

$$\Delta G(X)=-K_{B}T\;\ln\;P(X)$$

where *K*_*B*_ and *T* is the Boltzmann constant and absolute temperature, respectively. *P*(*X*) is the probability distribution of the molecular system along the PCs.

### 2.9. MM/PBSA calculations

The free energy of binding between protein and ligands was calculated using the Molecular Mechanics Poisson-Boltzmann Surface Area (MM/PBSA) method. This analysis was conducted with the gmx_MMPBSA tool, which processed the simulation trajectories generated through GROMACS. For accuracy, a stable 10 ns segment of these trajectories was extracted, with snapshots sampled every 0.01 ns. The calculation considered the complex’s internal energy, electrostatic solvation, and non-polar solvation contributions to determine the binding free energy (Δ*G*_binding_) as described by:

$${\Delta G}_{binding}=G_{complex}–(G_{receptor}+G_{ligand})$$

where Δ*G*_binding_ signifies the total binding energy of the protein-ligand complex, *G*_receptor_ signifies the free energy of the unbound protein and *G*_ligand_ signifies the free energy of the ligand.

## 3. Result

### 3.1. Molecular docking-based virtual screening

Molecular docking screening was performed to find the compounds that have high binding affinity to HDAC1. This was done from a list of repurposed drugs obtained from the DrugBank database of approved drugs. The screening process produced affinity scores and most likely docked poses for each compound in the library. This made it possible to get candidates with high binding affinity to HDAC1. After the screening process, compounds were further eliminated according to their ability to bind to HDAC1. The findings of the study revealed that the selected compounds had moderate to good binding affinity towards the binding pocket of HDAC1. However, the top ten out of 3600 compounds with the best binding affinity scores were ≤ −9.4 kcal/mol with HDAC1 (**Table 1**). While the reference HDAC1 inhibitor Pyroxamide showed a binding affinity of −5.9 kcal/mol. This proves that the chosen repurposed drugs are effective in targeting HDAC1 and can be recommended for further investigation in the development of repurposed HDAC1 inhibitors. As shown by the results of the binding affinity assay with HDAC1, these compounds are promising candidates that merit further examination for their inhibitory potential against HDAC1.

**Table 1:** Top 10 hits along with the reference inhibitor Pyroxamide with their docking scores towards HDAC1. Ligand Efficiency values are in kcal/mol/non-H atom.

S. No.	Drug	Binding Energy (kcal/mol)	pKi	Ligand Efficiency (kcal/mol/non-H atom)	Torsional Energy
1.	Bisdequalinium Chloride	−9.7	7.11	0.2205	0
2.	Aprepitant	−9.7	7.11	0.2622	2.4904
3.	Fosaprepitant	−9.7	7.11	0.2366	3.4243
4.	Zinostatin	−9.7	7.11	0.2021	3.4243
5.	Bagrosin	−9.6	7.04	0.4364	0.3113
6.	Dutasteride	−9.6	7.04	0.2595	1.2452
7.	Alectinib	−9.5	6.97	0.2639	0.9339
8.	Temoporfin	−9.5	6.97	0.1827	2.4904
9.	Florantyrone	−9.4	6.89	0.4087	1.5565
10.	Rifaximin	−9.4	6.89	0.1649	2.1791
11.	Pyroxamide	−5.9	4.33	0.3105	2.8017

### 3.2. Drug profiling and PASS evaluation

Considering the process of the selection of safe and effective molecules with the desired properties, it is crucial to study the biological properties of the hits. To this end, the process of drug profiling and the PASS analysis were used to study the drug-like properties of the identified hits. Among the 10 hits of the docking screening, one drug molecule, Alectinib, was chosen based on its drug profiling concerning anticancer activity. **Table 2** provides the biological activities and the degree of confidence in the prediction for Alectinib and the reference inhibitor Pyroxamide. According to the PASS analysis, Alectinib has good predictivity for antineoplastic, antimetastatic, and antioxidant activities. The obtained Pa values vary from 0,205 to 0,533. This suggests that Alectinib is likely to have these specific biological properties to a very large extent. The results predicted for Pyroxamide showed that it is an established HDAC1 inhibitor, which validates our prediction from the PASS server. These outcomes can be considered useful for understanding the possible pharmacological effects of Alectinib. Overall, the drug profiling and PASS activities for Alectinib indicated that it be further investigated and developed as a possible candidate for anticancer drug repurposing against HDAC1.

**Table 2:** Biological properties of the elucidated repurposed molecule and the refence inhibitor explored through the PASS webserver.

S. No.	Compound	Pa	Pi	Biological Activity
1.	Alectinib	0,533	0,049	Neurotransmitter uptake inhibitor
		0,349	0,126	Antineoplastic
		0,275	0,120	Myc inhibitor
		0,132	0,019	Insulin like growth factor 1 antagonist
		0,205	0,153	Antimetastatic
2.	Pyroxamide	0,726	0,002	Histone deacetylase inhibitor
		0,658	0,002	Histone deacetylase 1 inhibitor
		0,615	0,005	Tumor NF-alpha release inhibitor
		0,512	0,068	Antineoplastic
		0,216	0,008	Matrix metalloproteinase inhibitor

### 3.3. Interaction analysis

**Fig 1:**
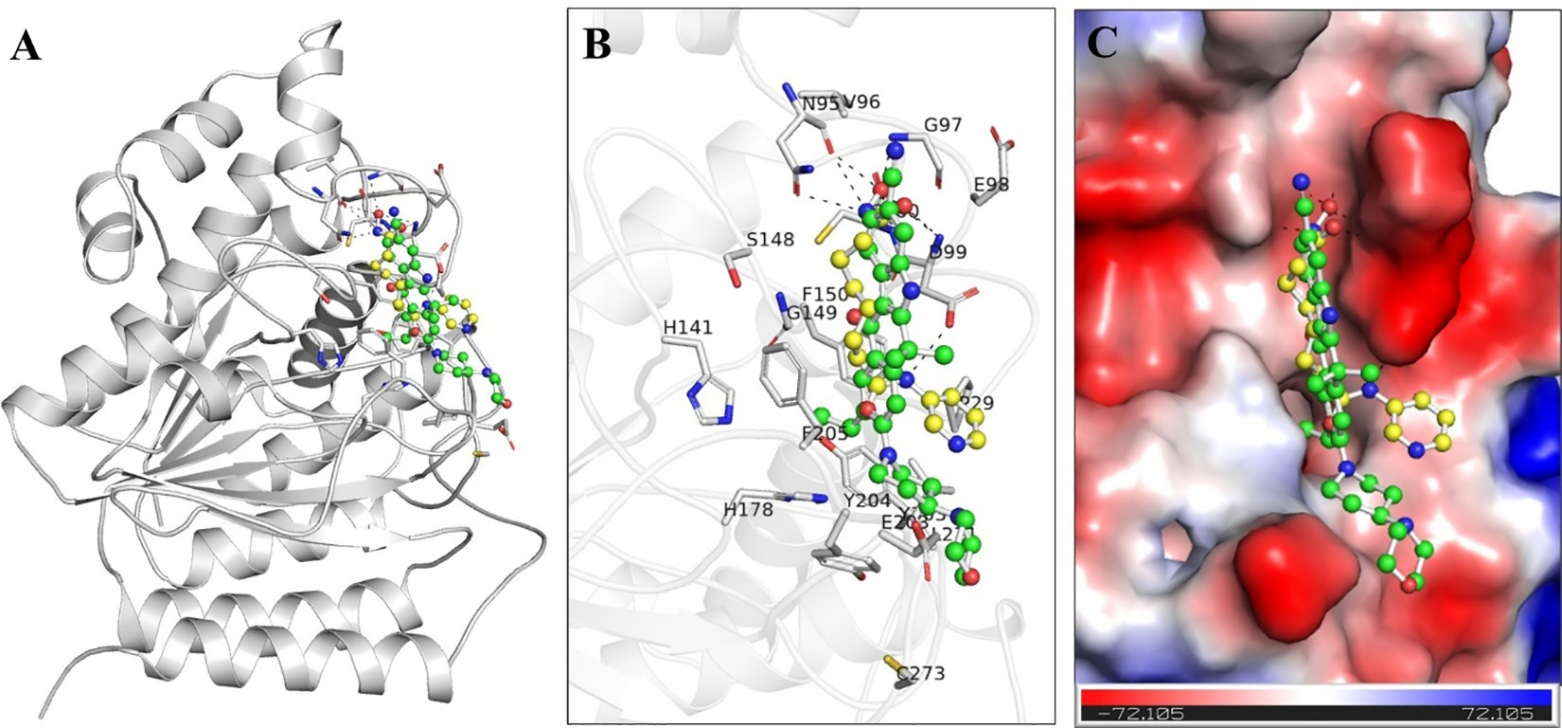
Interactions plots. (**A**) HDAC1 with Alectinib (green) and Pyroxamide. (**B**) Zoomed view of HDAC1 interactions with Alectinib and Pyroxamide. (**C**) The electrostatic potential of HDAC1 bound Alectinib and Pyroxamide.

The interactions between Alectinib and HDAC1 were examined using PyMOL and Discovery Studio visualizer which facilitated an understanding of their binding modes and interaction patterns (**Fig 1**). Notably, residues within the histone deacetylase domain of HDAC1, such as Asp99, His141, Gly149, Phe150, His178, Tyr204, Phe205, and Leu271, demonstrated significant interactions with docked Alectinib (**Fig 1A**). Particularly the active site His141 directly engaged with docked Alectinib (**Fig 1B**). This site is critical for HDAC1 activity for the critical and broad function of histone crotonylation in transcription [34, 35]. At the same time, the reference inhibitor Pyroxamide also shared various interactions with Alectinib. However, it doesn’t directly interact with the active site residue His141 of HDAC1. Both molecules were superimposed on each other and were observed to fit snugly within the binding pocket of HDAC1 (**Fig 1C**). This indicated favorable complementarity of the HDAC1 binding pocket and the compounds that further indicate their possible mechanism of inhibition. Interaction plots for the remaining nine compounds from the top ten hits, detailing their binding interactions with HDAC1, are provided in **S1 Fig** to give additional insights into their binding modes.

**Fig 2:**
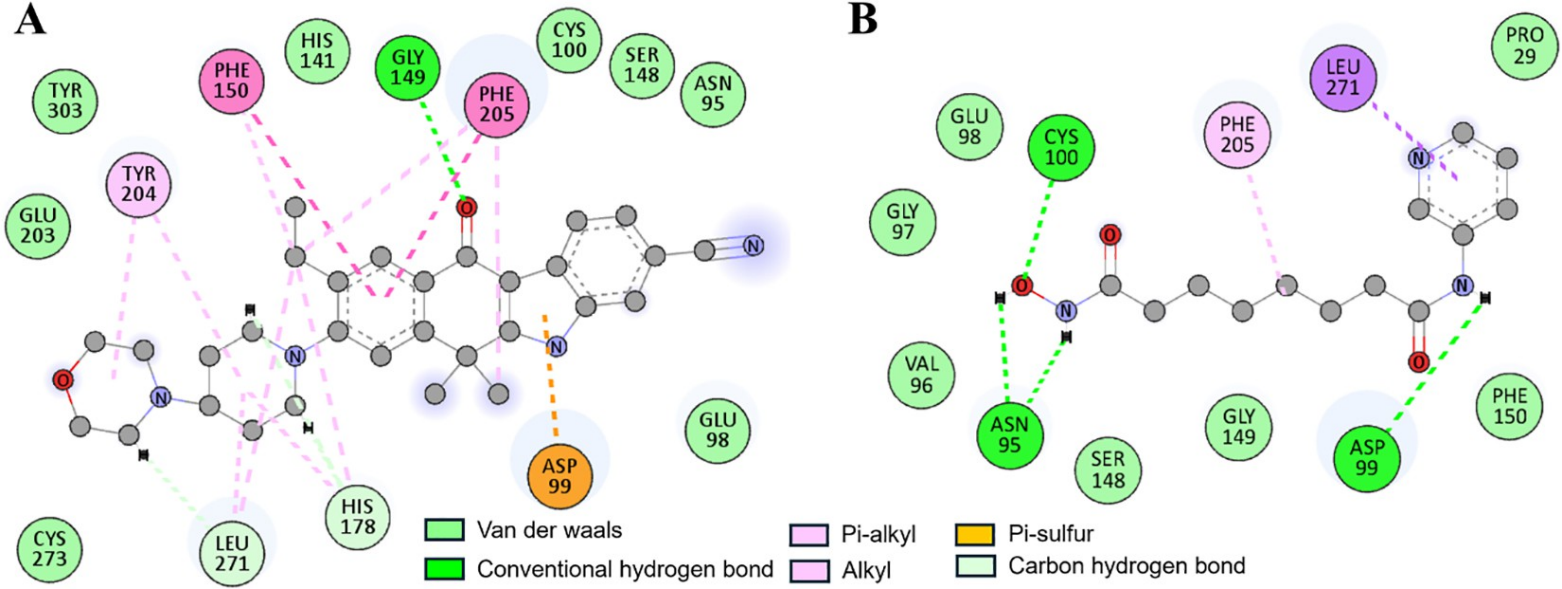
2D plots showing detailed interactions of (**A**) Alectinib and (**B**) Pyroxamide with HDAC1. The plots were generated through Discovery Studio Visualizer.

A closer examination indicated that both compounds bound to the active site pocket of HDAC1 where several residues contributed to the binding. The stability of the binding was kept through various close interactions including hydrogen bonds and hydrophobic interactions. The HDAC1-Alectinib complex showed stability through hydrogen bonds with Gly149, His178, and Leu271 and had several Pi-Pi stacked, Pi-alkyl, alkyl, and Van der Waals interactions (**Fig 2A**). Similarly, the HDAC1-Pyroxamide complex displayed interactions like hydrogen bonds with Asn95, Asp99, and Cys100 and Pi-sigma bond with Leu271, Pi-Alkyl bond with Phe205 along with several Van der Waals interactions (**Fig 2B**). The binding of Alectinib within the active site is quite promising, which may point to its use as a potential inhibitor of HDAC1. These findings provide insights into the molecular mechanisms that are responsible for the inhibitory activity of Alectinib on HDAC1.

### 3.4. MD simulations

#### 3.4.1. Structural deviations in HDAC1

**Fig 3:**
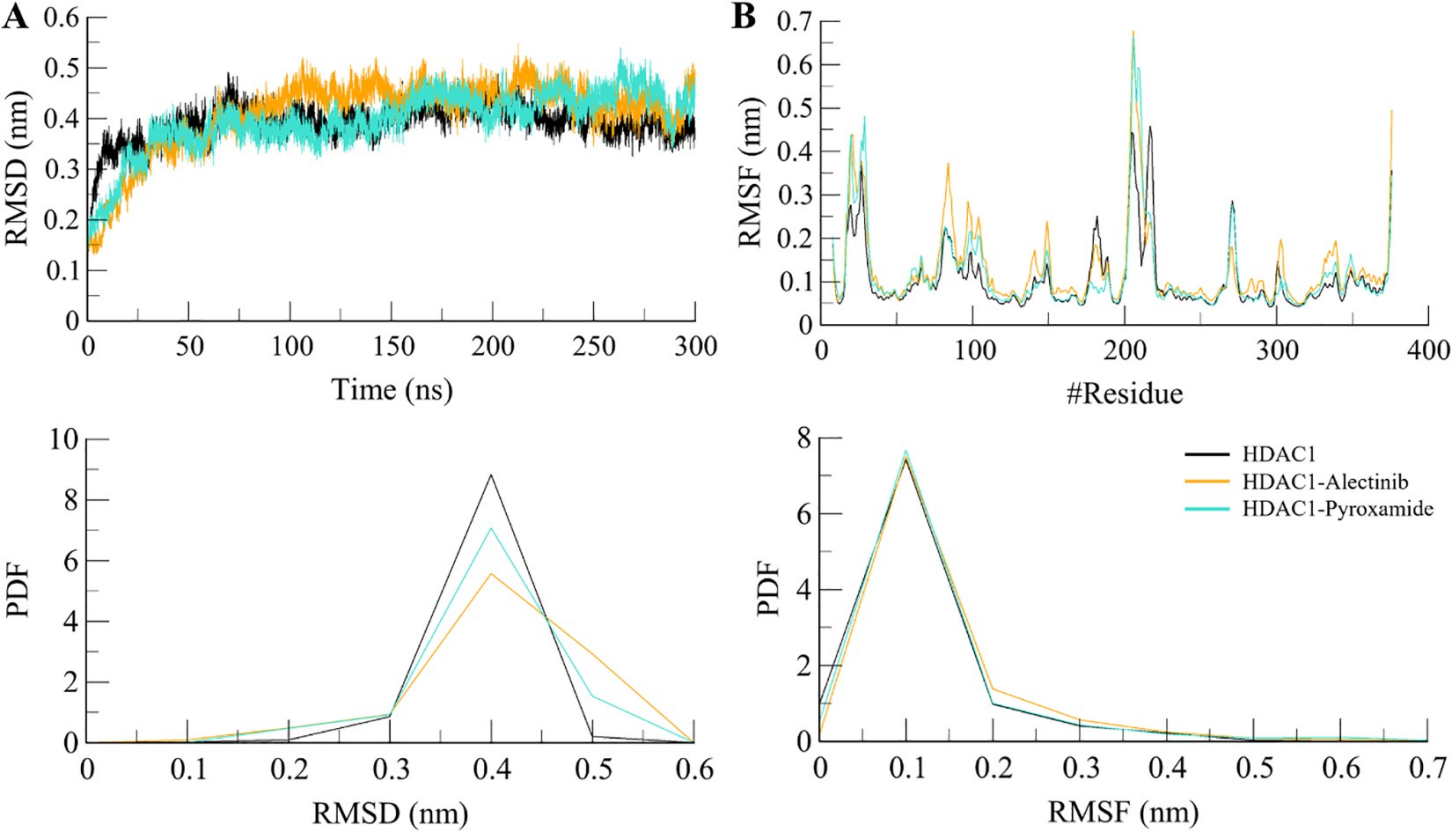
Structural dynamics of HDAC1 free (black), HDAC1-Alectinib (orange), and HDAC1-Pyroxamide (aqua). (**A**) RMSD and (**B**) RMSF plots after 300 ns of MD trajectories. Lower panels showed the probability distribution function (PDF) of the values.

Molecular docking studies give information on the interactions at the protein and ligand level at a particular time point [36]. However, knowing how these complexes behave in real life is crucial. Therefore, the MD simulation was performed on the complex of HDAC1 and the docked complexes of Alectinib and Pyroxamide. The objective was to understand the specificities of their interactions using various structural properties like RMSD and RMSF. The changes in these properties over the simulation time were investigated to evaluate structural changes in HDAC1 and its docked complexes during the simulation period (**Fig 3**). The changes in the RMSD values of all systems are presented in **Fig 3A**. These results show that they are stable and do not vary significantly during the 300 ns MD trajectories. RMSD values of the backbone atoms were applied to estimate the dynamic stability. Both complexes maintained the same level of stability for the entire simulation path without any structural switching.

Further, to analyze the stability of binding pocket residues during the MD simulation, RMSF for all the residues in HDAC1 was calculated. The RMSF analysis showed that HDAC1 had a similar pattern of residual fluctuations before and after ligand binding (**Fig 3B**). The residues in the vicinity of the ligand-binding site had comparatively low fluctuations as compared with the other residues. It pointed to the stability of the binding pocket in relation to the simulation. In summary, the RMSF in MD simulations revealed the proper stability of the HDAC1 and its complexes with Alectinib and Pyroxamide. The analysis of structural deviations gives useful information concerning the dynamics of the protein and protein-ligand complexes. It gives further insight into how they interact and the possible consequences in the process of drug discovery.

#### 3.4.2. Structural compactness in HDAC1

**Fig 4:**
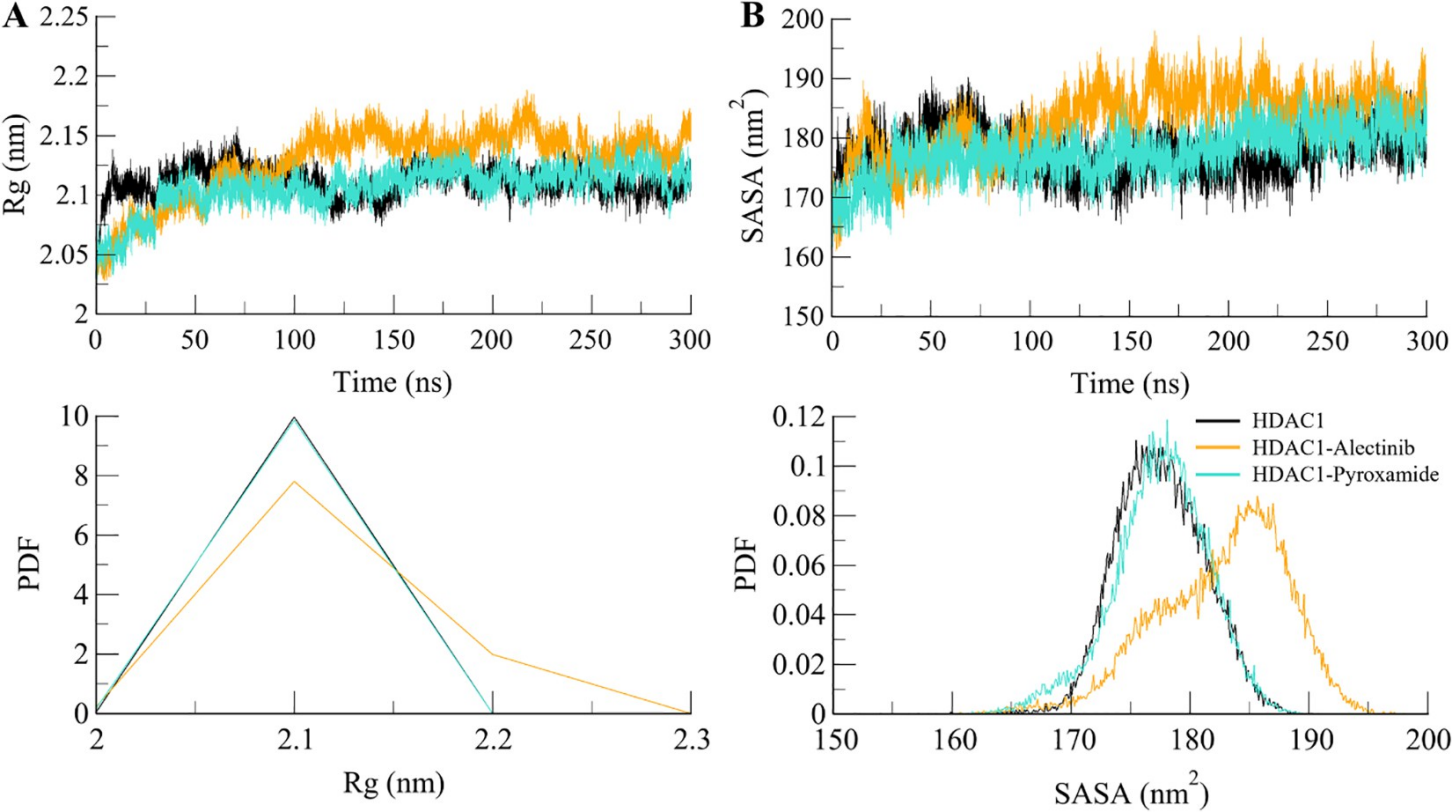
Structural compactness of HDAC1 free (black), HDAC1-Alectinib (orange), and HDAC1-Pyroxamide (aqua). (**A**) *R*g and (**B**) SASA plots after 300 ns of MD trajectories. Lower panels showed the probability distribution function (PDF) of the values.

The *R*g is used as a measure to analyze the folding and compactness of proteins and protein-ligand complexes [37]. The *R*g value increases with a decrease in the packing density of the structure, hence, a larger *R*g value denotes loose packing while a small *R*g value denotes tight packing. In MD studies, *R*g is used to determine the effect of ligand binding on protein conformational packing. The *R*g of each complex was calculated at the end of the simulation. This enabled us to investigate the impact of the compound binding on the conformational packing of HDAC1 (**Fig 4A**). The results showed that the *R*g value of HDAC1 was stable when it was bound to Alectinib. This suggested that there was a folded conformation of HDAC1 after the ligand binding in the simulation. The Alectinib binding to HDAC1 did not alter its compactness and therefore it indicates the stability of the complex.

To gain more insight into the folding/unfolding behavior of HDAC1 before and after the ligand binding, the time evolution of the SASA values was plotted during the entire simulation as shown in **Fig 4B**. SASA is the measure of the accessibility of each amino acid in a protein to the solvent [38]. SASA values that were shown in the present study followed the same trend as the *R*g values along the simulated time of 300 ns. After the ligand binding, the docked complexes were compact and stable during the simulation. At 100 ns, the SASA values of the HDAC1-Alectinib complex are increased slightly. This implies that some of the buried residues are exposed to the solvent while there are no major changes in the structure. In conclusion, the study of *R*g and SASA values gives an understanding of conformational changes in HDAC1 and its complexes with the ligands throughout the MD simulations.

#### 3.4.3. Dynamics of hydrogen bonds

**Fig 5:**
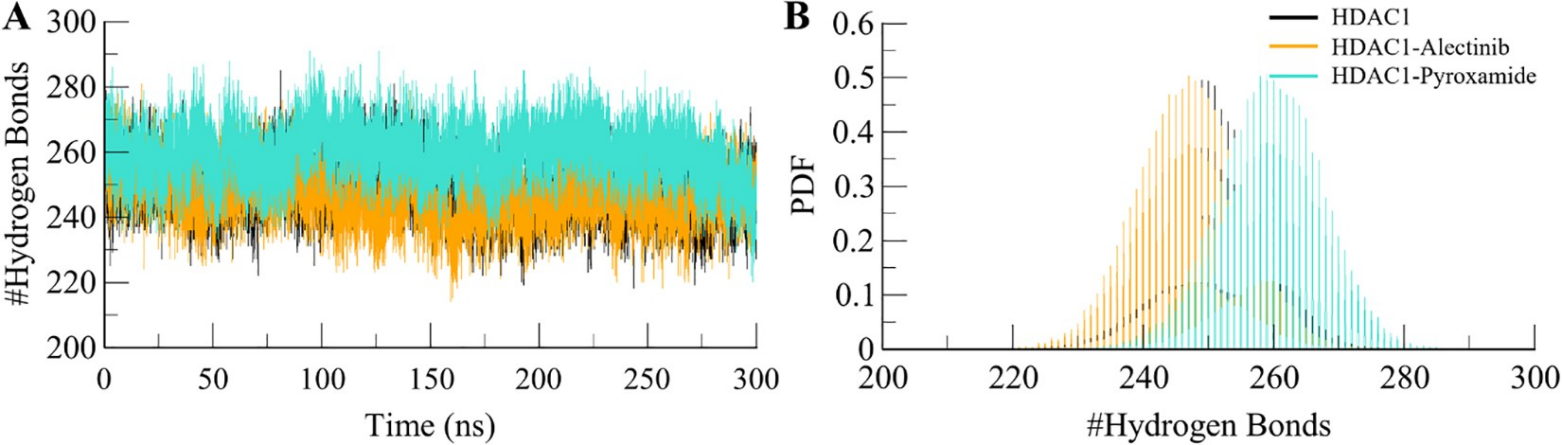
Intramolecular hydrogen bonds in HDAC1. **(A)** The dynamics of intramolecular hydrogen bonds in HDAC1 free (black), HDAC1-Alectinib (orange), and HDAC1-Pyroxamide (aqua). (**B**) The PDF of intramolecular hydrogen bonds in HDAC1.

Intramolecular hydrogen bonding is crucial for the stability of proteins since these bonds help in the formation of the structural framework of the protein [39]. The hydrogen bonds observed during MD simulation enable us to determine the impact of ligand binding on the protein. In this study, hydrogen bond analysis was carried out with a distance cutoff of 3.5 Å (**Fig 5**). The time evolution of intramolecular hydrogen bonds for all the systems was plotted and analyzed (**Fig 5A**). The plot showed that HDAC1 showed a fair consistency in the intramolecular hydrogen bonding thus maintaining its structural integrity during the simulations. The resulting PDF plot showed an overlapping pattern of hydrogen bond distribution (**Fig 5B**). This suggests that HDAC1 retained its structural integrity throughout the simulation after the binding of compounds.

**Fig 6:**
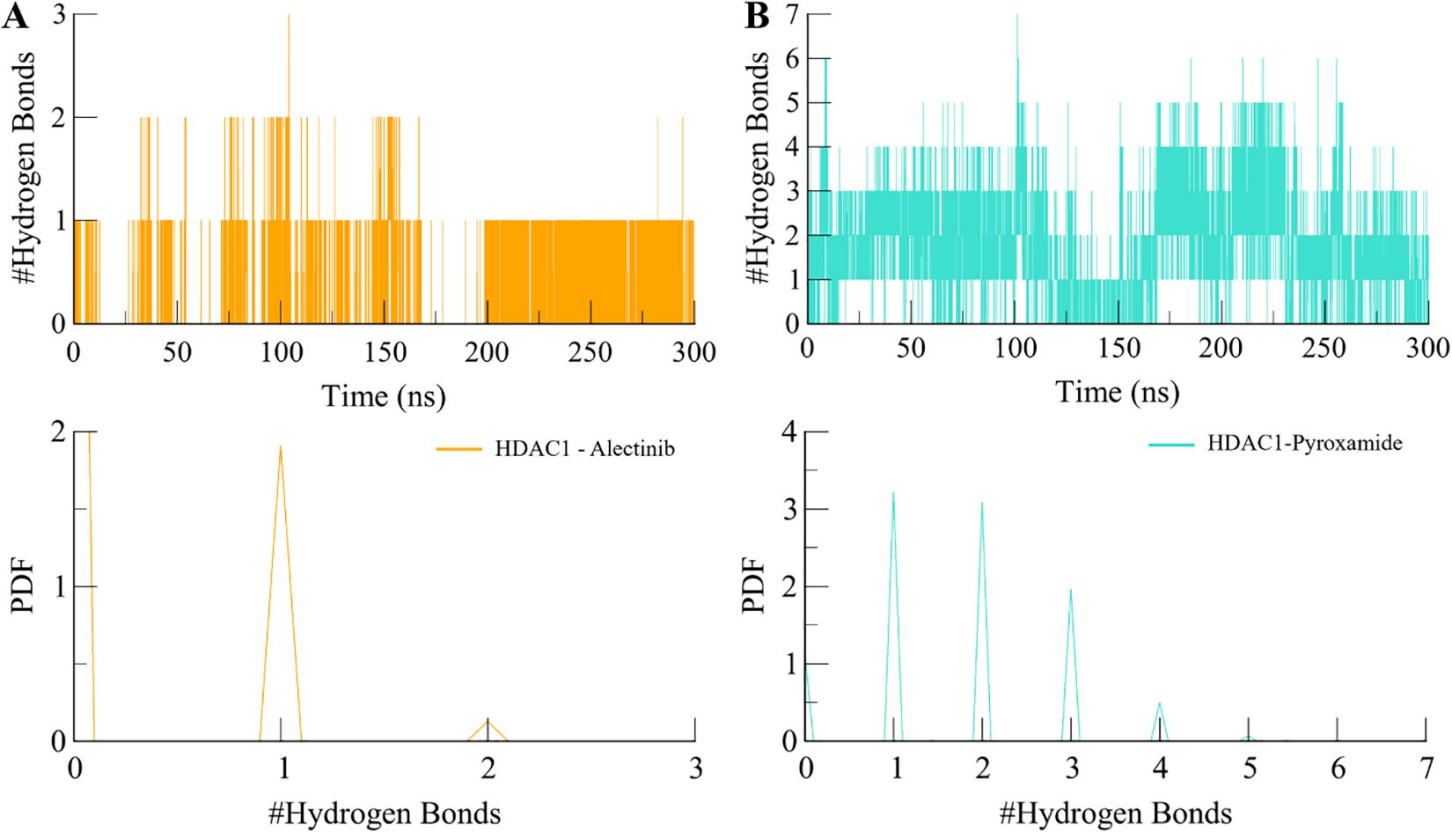
The dynamics of intermolecular H-bonds in HDAC1 and (A) Alectinib and (B) Pyroxamide. The lower panels show the PDF of intermolecular hydrogen bonds in HDAC1-ligands complexes.

Further, to explore the polarity of the interactions between the protein HDAC1 and the ligands Alectinib and Pyroxamide, the intermolecular hydrogen bonding analysis was conducted. The orientation and selectivity of these bonds are important for protein dynamics and the kinetic behavior of proteins as well as interactions with ligands [40]. The analysis in the present study used hydrogen bond analysis to reveal the molecular interactions that determine the stability of the HDAC1 complex in the presence of Alectinib and Pyroxamide (**Fig 6**). As for the hydrogen bonding, both ligands showed stability with slight fluctuation in their dynamical hydrogen bonding characteristics. In both cases, Alectinib formed a strong hydrogen bonding pattern with the HDAC1 protein. This helped to maintain its stability in the binding pocket for a long time as observed in the study. On the other hand, the dynamics of the hydrogen bonds between Pyroxamide and HDAC1 were found to be relatively higher compared to the previous case. The HDAC1-Alectinib complex formed up to three hydrogen bonds but maintained a stable bond throughout the simulation with minor fluctuations (**Fig 6A**). Likewise, the HDAC1- Pyroxamide formed complex had an average of 2 hydrogen bonds which at times was as high as seven bonds, but it maintained 2–3 bonds throughout the simulation (**Fig 6B**). As a result of further analysis using PDF plots, these observations were also corroborated, which showed that the intermolecular hydrogen bonds were also distributed evenly. Here, the higher frequency of one hydrogen bond in both complexes was observed (**Fig 6, lower panels**). These exciting results provide highly suggestive evidence of the fact that there are intermolecular hydrogen bonds between Alectinib and HDAC1 that maintained the protein-ligand complexes during the simulations.

### 3.5. Principal component analysis

**Fig 7:**
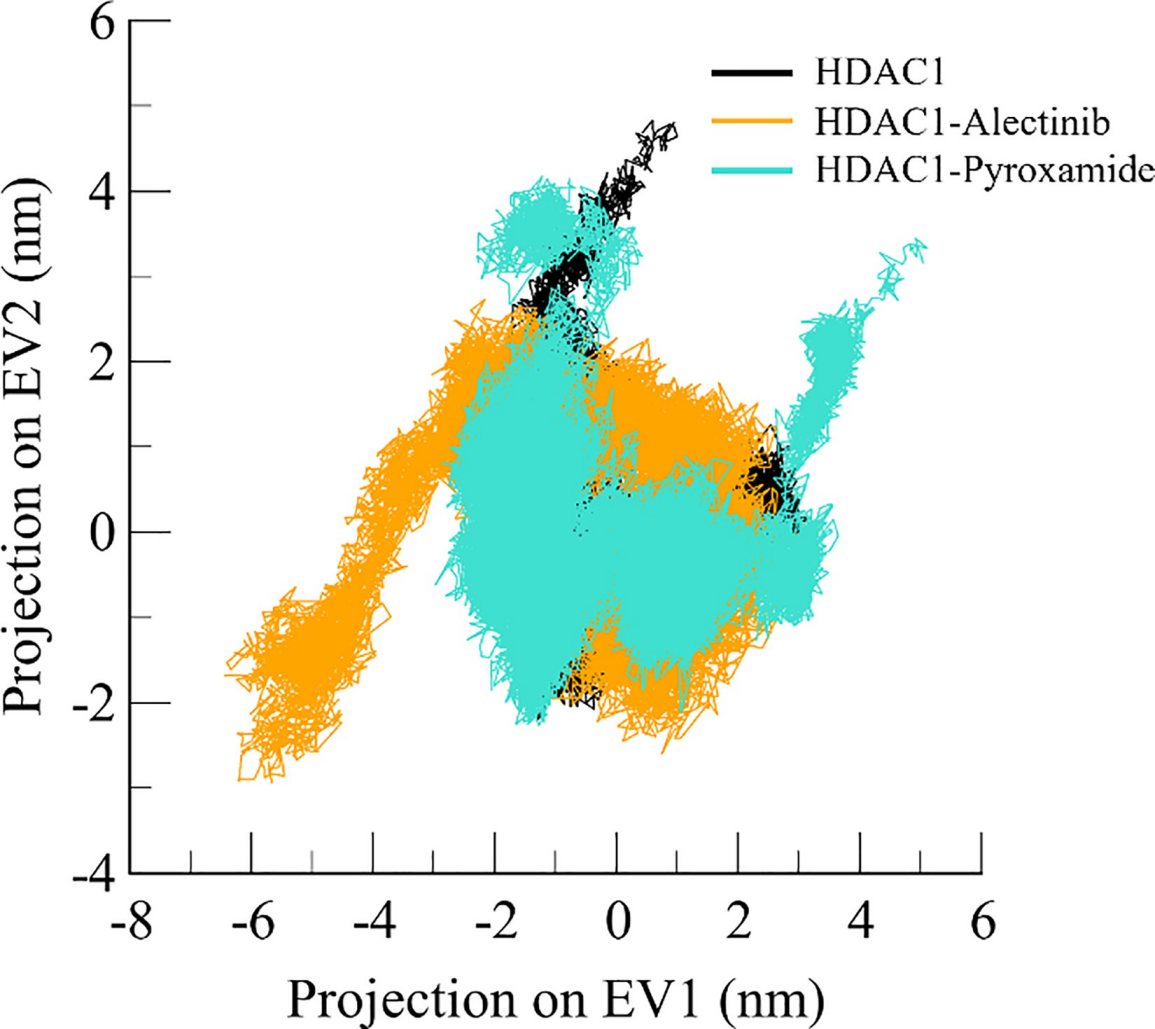
Conformational landscapes of HDAC1. 2D projection of HDAC1 free (black), HDAC1-Alectinib (orange), and HDAC1-Pyroxamide (aqua) complex calculated after 300 ns of MD trajectories.

PCA is a vital method for describing the general displacements of *C*α atoms in proteins [33]. These patterns are expressed as eigenvectors (EVs) obtained from the covariance matrix. In the current study, PCA was employed to analyze the directional changes of HDAC1 before and after Alectinib and Pyroxamide binding (**Fig 7**). The collective motions were analyzed by *C*α atom coordinates of HDAC1. Comparing the motions of the first two EVs, it can be observed that, in the case of the complex formation of HDAC1 with Alectinib, the motions are much more negative in comparison to free HDAC1 and the HDAC1-Pyroxamide complex. On the other hand, the motion of the HDAC1-Pyroxamide complex was relatively stable, like the free HDAC1. The plots indicated that for all the scenarios, the HDAC1-Alectinib complex was found to be in a larger phase space than both the free HDAC1 and the HDAC1-Pyroxamide. However, most of the conformational projections of the HDAC1-Alectinib complex are dense and aligned with the free HDAC1. It indicated that the binding of Alectinib might be involved in the stabilization of HDAC1.

### 3.6. Free energy landscape analysis

**Fig 8:**
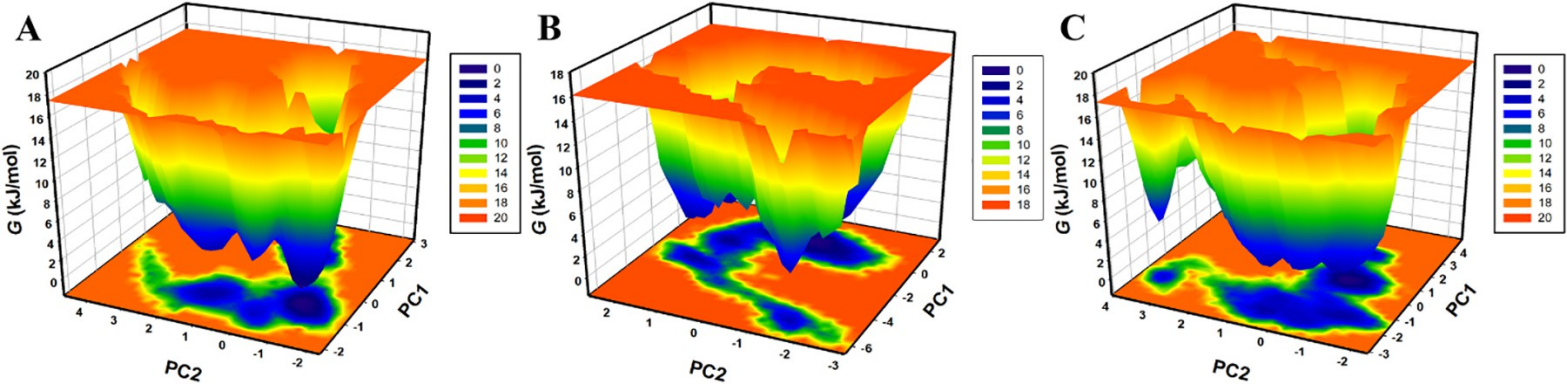
Free energy landscapes of HDAC1. 3D FEL of (**A**) HDAC1 free, (**B**) HDAC1-Alectinib, and (**C**) HDAC1-Pyroxamide.

The FELs were generated based on the first two PCs to classify the conformational states of HDAC1 and its complexes with ligands (**Fig 8**). FEL analysis provides information about the folding stability of the HDAC1 and different metastable states of the protein before and after ligand binding. As shown in **Fig 8A**, HDAC1 has a unique single global energy minimum with two basins as the conformations of the protein. On the other hand, the HDAC1-Alectinib complex has a wider global energy minimum profile with a transition state in addition to multiple native energy minima (**Fig 8B**). This transition state is similar to the free HDAC1 structure but with small deviations. In the case of the HDAC1-Pyroxamide complex, the native basin of HDAC1 goes out to relative energy minima of greater breadth, suggesting several conformations of the protein within a small region (**Fig 8C**). These results draw attention to the conformational states of the HDAC1 and its ligand complexes and the impact of ligand binding on the energy profile. The enlarged energy minima in the HDAC1-ligand complexes suggest that the structures are more flexible and have multiple conformations that are energetically favorable, which is consistent with the complex dynamics involved in ligand binding.

### 3.7. MM/PBSA analysis

The binding free energy of HDAC1 protein-ligand complexes was analyzed using the MM-PBSA method to assess the thermodynamic stability of the interactions. This parameter reflects the energetic changes accompanying complex formation, indicating interaction strength. Table 3 presents the binding free energy components along with their standard deviations. The analysis demonstrates that both HDAC1-Alectinib and HDAC1-Pyroxamide complexes show stable and favorable binding energies, suggesting robust protein-ligand associations. These stable interactions highlight Alectinib’s promise as a potential HDAC1 inhibitor for cancer therapy, underscoring its therapeutic relevance.

**Table 3:** Binding free energy calculations for HDAC1 complexes via MM-PBSA analysis.

Complex	Δ*G*_VDWAALS_	Δ*E*_EL_	Δ*E*_PB_	Δ*E*_NPOLAR_	Δ*G*_GAS_	Δ*G*_SOLV_	Δ*G*_Total (kJ/mol)_
HDAC1-Alectinib	−25.77	−21.91	28.12	−2.82	−47.67	25.30	−22.38 ± 4.25
HDAC1-Pyroxamide	−26.49	−0.45	15.09	−2.95	−26.94	12.14	−14.80 ± 2.58

## 4. Discussion

HDAC1 belongs to Class I of HDACs and plays a critical role in gene regulation and is directly associated with carcinogenesis and neurodegenerative diseases [41]. In this study, Alectinib, an ALK inhibitor previously approved by FDA, is considered as a potential repurposed drug targeting HDAC1. The findings demonstrate the ability of Alectinib to serve as a potential HDAC1 repurposed inhibitor through virtual screening, molecular docking, MD simulations, and MM-PBSA binding free energy estimations. The results reveal that Alectinib interacts more potently with HDAC1 than the reference inhibitor Pyroxamide based on the docking scores and stable MD simulation. In prior works, HDAC inhibitors have shown the ability to exert cytostatic effects on cancer cells, as well as lead to apoptosis and differentiation through mechanisms that involve modifications of histone acetylation and chromatin remodeling [11, 16]. The binding residues of Alectinib depicted in this study, including His141 and Asp99, are in concordance with the binding profiles of other known HDAC inhibitors, which supports the evaluation of Alectinib as a repurposing candidate [35].

Notably, the MD simulations provide more evidence for the structural stability of the Alectinib-HDAC1 complex, which experienced small structural fluctuations and maintained strong hydrogen bonds. These findings are in parallel with other studies in which stability in the HDAC binding pocket was a factor indicative of the inhibitory effects [14]. The binding energy results from our MM-PBSA calculations gave us additional information that supported our hypothesis of Alectinib being a solid HDAC1 inhibitor with a stable protein-ligand complex. There are several significant benefits for repurposing Alectinib for HDAC1 inhibition, including Alectinib’s prior safety and pharmacokinetic profiles, as well as bioavailability [17]. This approach could help fast-track the development of Alectinib for potential use in targeting HDAC1, which has drawbacks often seen in the creation of novel HDAC inhibitors, including off-target effects, toxicity, and resistance. Furthermore, Alectinib showed a dual inhibition effect on both ALK and HDAC1, and therefore, it may have additional therapeutic value in cancer subtypes involving ALK and HDAC1, such as ALK-rearranged and HDAC1-overexpressing tumors.

Although Alectinib shows favorable binding and selectivity for HDAC1, it is crucial to consider its full spectrum activity and possible side effects in its repositioning therapy. Alectinib was originally developed to inhibit ALK in non-small cell lung cancer (NSCLC), and the inhibition of other kinases may explain additional biological properties of HDAC1. To confirm the selectivity and the efficacy of Alectinib as a selective HDAC1 inhibitor, further work should be done to investigate the interactions of the compound with other isoforms of HDACs and kinases to exclude the possible cross-reactivity that may affect the safety and efficacy of the compound [42]. Despite the exciting development of HDAC inhibitors, the development of resistance to these drugs continues to be a major problem in cancer treatment. However, this study suggests that Alectinib may help stabilize HDAC1, although future studies should focus on the potential resistance mechanisms of HDAC inhibition. There are several strategies by which cancer cells can develop resistance to HDAC inhibitors, such as HDAC isoform compensation, an increase in efflux transporters, and changes in drug metabolism [13]. The fact that Alectinib is an ALK inhibitor with added properties of HDAC1 inhibition may offer a dual targeting mechanism that can potentially overcome these resistance pathways, although this hypothesis should be tested.

While the findings provide robust computational evidence supporting Alectinib’s potential as an HDAC1 inhibitor, the study is inherently limited by the lack of experimental validation. As a purely *in silico* investigation, additional *in vitro* and *in vivo* studies are essential to confirm Alectinib’s HDAC1 inhibitory activity, cytotoxicity, and selectivity in cancer models. Future research should also focus on elucidating the full pharmacological profile of Alectinib in the context of HDAC inhibition, examining its binding affinity across different HDAC isoforms, and assessing its ability to cross the blood-brain barrier. This aspect is particularly relevant given HDAC1’s role in neurodegenerative diseases, suggesting potential applicability beyond oncology. In addition, structural modifications to Alectinib may enhance its specificity for HDAC1, potentially reducing off-target effects while retaining its inhibitory activity. Ultimately, this study contributes to the growing field of drug repurposing, highlighting how existing drugs can be leveraged to target new pathways in cancer and potentially neurodegenerative diseases, thereby expediting the development of effective therapies.

## 5. Conclusions

Targeting HDAC1 through repurposed drugs is a promising strategy for therapeutic development in modern drug discovery against cancer and neurodegeneration. This study has given a new perspective on the relationship between HDAC1 and Alectinib as its repurposed inhibitor molecule. The virtual screening, molecular dynamics, and conformational dynamics studies such as RMSD, RMSF, *R*g, SASA, hydrogen bonding, secondary structure, and PCA confirmed that Alectinib could be repurposed for targeting HDAC1. In particular, the interaction between Alectinib and HDAC1 involves the formation of strong hydrogen bonds that ensure the stability of the complex. Evaluation of the all-atom MD simulation trajectories showed that the binding of Alectinib led to the increase in compactness and stability of its docked complex with HDAC1. The PCA and FEL analyses, along with the MM-PBSA, further revealed that the binding of Alectinib elicited different conformational motions with reasonable stability. Taken together, the results of this study may be useful for the further investigation of the repurposing of existing drugs and the development of new drugs similar to them as potential HDAC1 inhibitors. They form the foundation for other studies to determine other therapies for targeting epigenetic modulators in the progression of cancer. Therefore, the present investigation leads to the hypothesis that Alectinib has a high potential for HDAC1-related carcinogenesis after validation. Further experimental studies, including *in vitro* and *in vivo* evaluations, are essential to confirm the efficacy and safety of Alectinib in HDAC1-associated cancer therapeutics. These studies will help validate the computational predictions and determine the therapeutic potential of Alectinib as an HDAC1 inhibitor in anticancer therapeutic development.

## Supporting information

S1 Fig 2D Interaction plots for the remaining nine compounds from the top ten hits against HDAC1. (PPTX)
