# Supplementary material for: Virtual screening and molecular dynamics simulations identify repurposed drugs as potent inhibitors of Histone deacetylase 1: Implication in cancer therapeutics
Source: PLoS One. 2025 Jan 3;20(1):e0316343. doi: 10.1371/journal.pone.0316343 (PMC11698309; doi:10.1371/journal.pone.0316343)
Supplement: S1 Fig — (PPTX) [file pone.0316343.s001.pptx]

## Slide 1
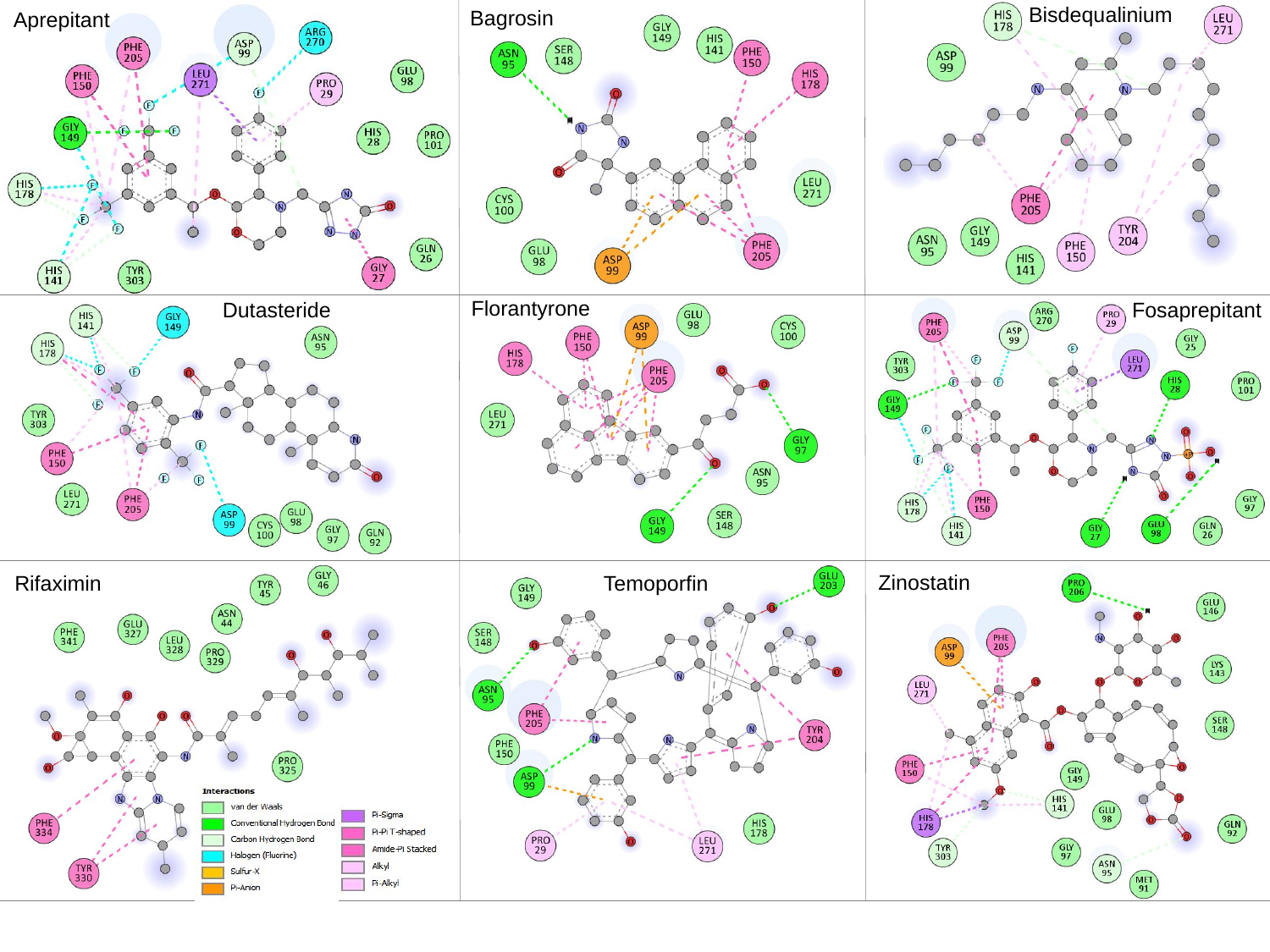

Aprepitant
Bisdequalinium
Bagrosin
Florantyrone
Dutasteride
Fosaprepitant
Zinostatin
Temoporfin
Rifaximin
